# Distinct profiles of cerebral oxygenation in focal vs. secondarily generalized EEG seizures in children undergoing cardiac surgery

**DOI:** 10.3389/fneur.2024.1353366

**Published:** 2024-05-09

**Authors:** Rouyi Lin, Na Du, Shuyao Ning, Mingjie Zhang, Jinqing Feng, Xinxin Chen, Li Ma, Jia Li

**Affiliations:** ^1^Guangdong Provincial Key Laboratory of Research in Structural Birth Defect Disease, Guangzhou Women and Children’s Medical Center, Guangzhou Medical University, Guangzhou, China; ^2^Clinical Physiology Laboratory, Institute of Pediatrics, Guangzhou Women and Children’s Medical Center, Guangzhou Medical University, Guangzhou, China; ^3^Heart Center, Guangzhou Women and Children’s Medical Center, Guangzhou Medical University, Guangzhou, China; ^4^Department of Electroneurophysiology, Guangzhou Women and Children’s Medical Center, Guangzhou Medical University Guangdong Province, Guangzhou, China; ^5^Department of Radiology, Guangzhou Women and Children’s Medical Center, Guangzhou Medical University Guangdong Province, Guangzhou, China

**Keywords:** cardiopulmonary bypass, focal seizures, secondarily generalized seizures, cerebral oxygen saturation, brain injuries

## Abstract

**Objectives:**

Seizures are common in children undergoing cardiopulmonary bypass (CPB). Cerebral oxygen saturation (ScO_2_) by near-infrared spectroscopy is routinely monitored in many centers, but the relations between the levels and changes of ScO_2_ and brain injuries remain incompletely understood. We aimed to analyze the postoperative profiles of ScO_2_ and cerebral blood flow velocity in different types of EEG seizures in relation to brain injuries on MRI.

**Methods:**

We monitored continuous EEG and ScO_2_ in 337 children during the first 48 h after CPB, which were analyzed in 3 h periods. Cerebral blood flow peak systolic velocity (PSV) in the middle cerebral artery was measured daily by transcranial Doppler. Postoperative cerebral MRI was performed before hospital discharge.

**Results:**

Based on the occurrence and spreading types of seizures, patients were divided into three groups as patients without seizures (Group N; *n* = 309), those with focal seizures (Group F; *n* = 13), or with secondarily generalized seizures (Group G; *n* = 15). There were no significant differences in the onset time and duration of seizures and incidence of status epilepticus between the two seizures groups (*Ps* ≥ 0.27). ScO_2_ increased significantly faster across Group N, Group G, and Group F during the 48 h (*p* < 0.0001) but its overall levels were not significantly different among the three groups (*p* = 0.30). PSV was significantly lower (*p* = 0.003) but increased significantly faster (*p* = 0.0003) across Group N, Group G, and Group F. Group F had the most severe brain injuries and the highest incidence of white matter injuries on MRI among the three groups (*Ps* ≤ 0.002).

**Conclusion:**

Postoperative cerebral oxygenation showed distinct profiles in secondarily generalized and particularly focal types of EEG seizures in children after CPB. A state of ‘overshooting’ ScO_2_ with persistently low PSV was more frequently seen in those with focal seizures and more severe brain injury. Information from this study may have important clinical implications in detecting brain injuries when monitoring cerebral oxygenation in this vulnerable group of children after CPB.

## Introduction

Acquired brain injuries and neurodevelopmental impairment are common and potentially devastating comorbidities in children with congenital heart disease (CHD) undergoing CPB (1–3). Studies using continuous electroencephalographic (EEG) monitoring have reported that seizures occurred in 5–20% of patients during early postoperative period and was a marker for acute brain injury and associated with worse neurodevelopmental outcomes (1, 2, 4–6). According to the International League Against Epilepsy, seizures included focal and initially focal then secondarily generalized types depending on whether seizures widespread bilaterally or not (7). We and others have previously reported that among patients with seizures, the focal seizures occurred in 20–70% of patients and secondarily generalized type occurred in 15–70% (2, 4, 5). In addition to seizures, the EEG abnormal discharges also included some micro-scale patterns, such as spikes/sharp waves, which may occur when cerebral perfusion and oxygen supply are too limited to manifest seizures which demand much energy and oxygen supply (8–10). These micro-scale patterns have been related to neurologic risk conditions, e.g., neonatal asphyxia (11), but have not been reported in children after cardiac surgery.

The early postoperative period in children after CPB is characterized by the profound imbalance of systemic and cerebral oxygen transport with increased oxygen consumption and decreased oxygen delivery (12–14). It has been further reported that imbalanced systemic oxygen transport has important influences on early postoperative cerebral oxygen saturation (ScO_2_) (12). Cerebral metabolic rates and energy use may increase several times during seizures, thus worsening cerebral oxygenation status (8–10, 15). Nonetheless, the alteration of cerebral oxygenation and its relation with EEG abnormalities during the early post-CPB period remain largely unexplored.

Currently, near-infrared spectroscopy (NIRS), transcranial Doppler (TCD), EEG, and magnetic resonance imaging (MRI) are generally used to assess cerebral oxygenation and brain functional and anatomical injuries (1–6, 16–21). NIRS is routinely used in many centers to continuously monitor ScO_2_ which is the equilibrium of oxyhemoglobin and deoxyhemoglobin in a mixture of veins, arteries, and capillaries in the underlying tissue and reflects a regional state of oxygenation (16–20). Early postoperative low ScO_2_ has been reported to be associated with brain injury and neurodevelopmental impairment (18–20). But the clinical implications of its changes and the not uncommonly observed high levels of ScO_2_ during the early post-CPB period are incompletely understood. From ScO_2_ and systemic arterial oxygen saturation (SaO_2_), cerebral oxygen extraction ratio (CERO_2_) can be calculated, manifesting cerebral oxygen consumption relative to oxygen delivery. TCD measures cerebral blood flow velocity manifesting cerebral oxygen delivery (21).

Therefore, we aimed to investigate the profiles of cerebral oxygenation parameters in different types of EEG seizures and discharge abnormalities and to propose the potential pathophysiological mechanisms and clinical implications using these techniques.

## Patients and methods

### Patients

**Figure 1:**
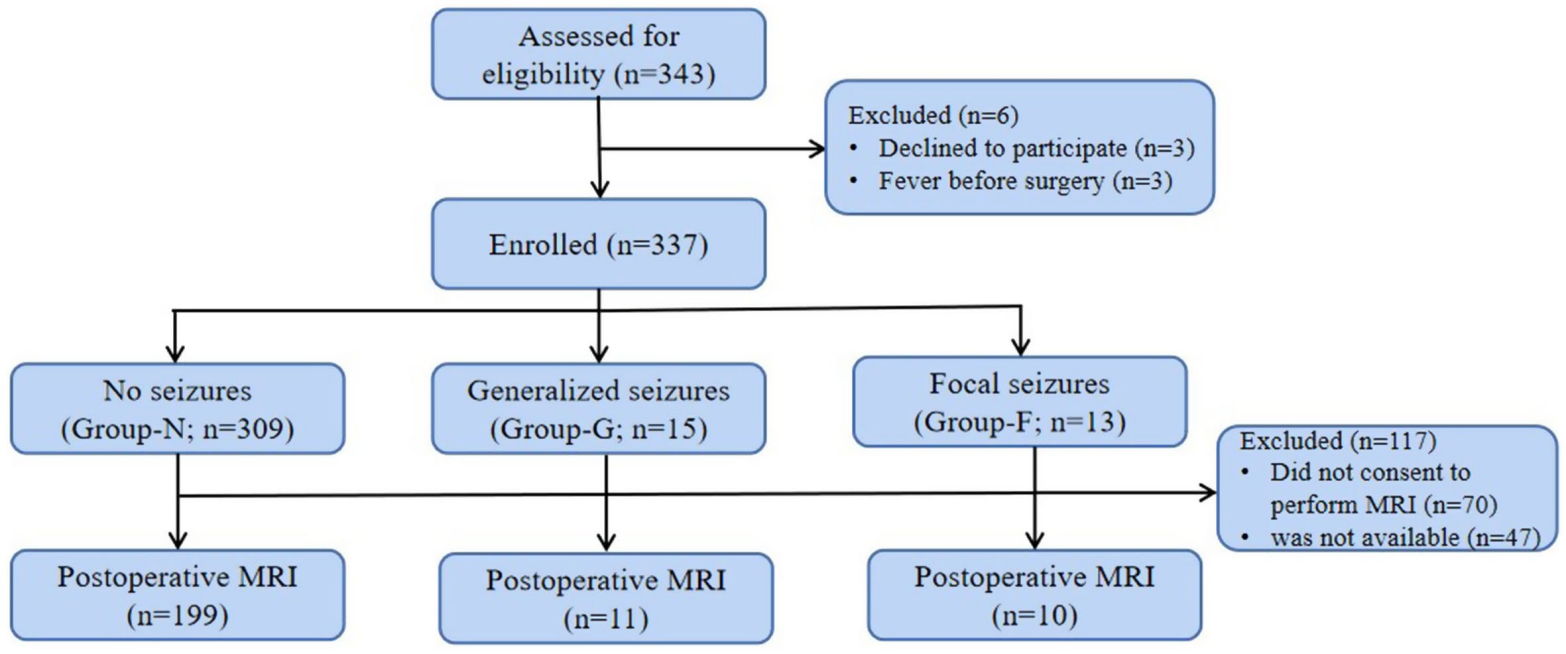
Flow chart of patient populations undergoing EEG and MRI assessment.

After the institutional ethics approval (No. 46201) and informed consent obtained at the Guangzhou Women and Children’s Medical Center, a total of 337 patients were enrolled from January 2019 to December 2021 (Figure 1). The most complex CHD patient on the daily surgical list was screened to be approached for recruitment according to STS-EACTS (the Society of Thoracic Surgeons-European Association for Cardio-Thoracic Surgery) Mortality Categories (4, 22). Patients with the recognizable syndrome of congenital anomalies, previous CPB, scalp vein puncture, postmenstrual age < 37 weeks, cerebral hemorrhage by Doppler ultrasound were excluded.

### Intraoperative procedures and postoperative managements

#### Intraoperative procedures

Patients were premedicated with 0.01 mg/kg penehyclidine hydrochloride. Anesthesia was induced with propofol (2–3 mg/kg), cis-atracurium (0.2–0.3 mg/kg), and sufentanil (0.5–1 μg/kg). It was maintained with sevoflurane (2–3%), dexmedetomidine (0.3–1 μg/kg/h). Standard cardiopulmonary bypass as described elsewhere (4). A bolus of heparin (500 units/kg) was administered to maintain activated clotting time of whole blood >480 s. Deep hypothermic circulatory arrest (DHCA) was performed without antegrade selective cerebral perfusion for the repair of aortic arch obstructive abnormalities.

#### Postoperative managements

Standard postoperative management was used as described elsewhere (4). Patients receive time-cycled pressure control/pressure support ventilation on arrival in the CICU, followed by non-invasive ventilation when appropriate. Sedation consisted of continuous intravenous infusion of sufentanil (0.2 μg/kg/min) and dexmedetomidine (2 μg/kg/min) and intermittent administration of midazolam (0.1 mg/kg). Inotropic and vasoactive drugs included dopamine, milrinone and epinephrine to maintain arterial blood pressure (systolic pressure 60–90 and 90–105 mmHg in neonates and children, respectively).

### Methods of measurements

All patients had monitoring of heart rate, arterial blood pressure, rectal temperature, and SaO_2_.

#### EEG monitoring

Continuous Video-EEG was recorded using Nicolet monitor (CareFusion, Middleton, Wisconsin, United States). The recording of electrical activity was collected from scalp electrodes positioned in the FP1, C3, T3, O1, Cz, FP2, C4, T4, and O2 positions according to the international 10–20 system. Electrographic seizures was defined as epileptiform discharges averaging >2.5 Hz for ≥10 s, and status epilepticus as continuous seizures ≥10 min or for a total duration of ≥20% of any 60 min period of recording (23). The origin of seizures indicates the region of the initial seizure onset. Focal seizures was defined as localized and limited to one hemisphere (either a left-or right-sided lateralization) and secondarily generalized seizures was defined as initially focal then spreading to bilaterally diffused (7). Spikes/sharp waves were defined as high amplitude (≥2.5 times of background voltage) and short duration (<200 ms) (23). All EEGs were analyzed in 3 h periods by the qualified technicians (RYL and SYN) independently and finalized by SYN.

#### Cerebral oxygen saturation

ScO_2_ was continuously measured using NIRS (INVOS 5100C, Medtronic & Covidien, Troy, MI, United States) (12). The sensors were placed on the children’s forehead below the hairline to the right and left of the midline and recorded every 3 h. Averaged bilateral ScO_2_ was used for Group N and Group G. In Group F, ipsilateral ScO_2_ to the onset of seizures and the other side of the brain were separately analyzed. CERO_2_ was calculated using the following equation: (SaO_2_ − ScO_2_)/SaO_2_.

#### Cerebral blood flow velocity

PSV of the middle cerebral artery was measured with TCD with a 2 MHz pulse-wave ultrasound transducer, which was fixed above the zygomatic arch (Multi-Dop T; DWL Elektronische Systeme GmbH, Sipplingen, Germany) and interrogated the portion of the middle cerebral artery near its junction with the anterior cerebral artery (21). PSV was recorded on postoperative day 0, day 1, and day 2. Bilateral PSV was averaged for Group N and Group G. In Group F, we analyzed separately PSV ipsilateral to the onset of seizures and the other side of the brain.

#### Cerebral MRI

MRI scans were performed on a 3 T Magentom Prisma scanner (Siemens, Munich, Germany) including standard T1, T2, diffusion-weighted imaging, and diffusion-tensor imaging at the median 9 (3–37) days after surgery. Brain injuries included white matter injury, stroke, and hemorrhage and were graded as mild, moderate, and severe using the standard method (24). All MRIs were evaluated by a pediatric neuroradiologist (MJZ).

#### Clinical data

Demographic data, STS-EACTS Mortality Categories (22), the duration of postoperative mechanical ventilation, CPB and aortic cross-clamp (ACC), Deep hypothermic circulatory arrest (DHCA), CICU, hospital stay, and death were collected (Table 1).

**Table 1:** Demographic and perioperative data in the study cohort.

Variable	Group N (*n* = 309)	Group G (*n* = 15)	Group F (*n* = 13)	*p*-value
Gender				>0.99
Male, *n* (%)	184 (60)	9 (60)	8 (62)	
Female, *n* (%)	125 (40)	6 (40)	5 (38)	
Age (d)	100 (1–354)	78 (1–196)	41 (1–225)	0.09
Weight (kg)	4.8 (2–9.5)	4.6 (3.4–9)	3.9 (2.8–6.2)	0.09
BSA (m^2^)	0.28 (0.16–0.44)	0.27 (0.22–0.41)	0.24 (0.20–0.33)	0.10
STS-EACTS Mortality Category, *n* (%)				<0.0001
1	86 (27.8)	3 (20.0)	1 (7.7)	
2	102 (33.0)	6 (40.0)	3 (23.1)	
3	65 (21.0)	5 (33.3)	5 (38.5)	
4	56 (18.2)	1 (6.7)	4 (30.7)	
CPB time (min)	106 (28–334)	131 (80–289)	167 (101–270)	<0.0001
ACC time (min)	55 (0–249)	78 (42–141)	74 (55–166)	<0.0001
DHCA, *n* (%)	53 (17.2)	2 (13.3)	8 (61.5)	0.001
DHCA time (min)	18 (13–42)	15 (15–15)	18 (13–35)	0.19
Postoperative mechanical ventilation time (h)	49 (7–1,045)	53 (12–1,218)	46 (20–713)	0.89
CICU stay (day)	5 (1–159)	7 (2–99)	5 (2–45)	0.12
Hospital stay (day)	12 (4–164)	14 (7–99)	13 (7–46)	0.07
Death, *n* (%)	8 (2.8)	0 (0.0)	0 (0.0)	>0.99

BSA, body surface area; STS-EACTS, The Society of Thoracic Surgeons-European Association for Cardio-Thoracic Surgery; CPB, cardiopulmonary bypass; DHCA, deep hypothermic cardiac arrest; ACC, aortic cross-clamping; CICU, cardiac intensive care unit.

### Statistical analysis

Data were described as median (range) or frequency (%) when appropriate. Comparisons of parameters across the three groups were made using the Kruskal–Wallis test for non-normal distribution variables and Chi-squared or Fish’s exact test for categorical variables when appropriate. Comparisons of seizures details between Group G and Group F were made using the Mann–Whitney U-test for non-normal distribution variables and the Chi-squared test for categorical variables. Mixed linear regression for repeated measures was used to analyze the profiles of variables. It was also used to compare the differences in levels and trends between groups with analysis of the effects of group interaction between time and group. The parameter estimates and probability values of the group effect (*P*_group_) indicate the difference in the overall levels of each variable between the groups and the interaction of time and group (*P*_group × time_) indicates the difference in trends of each variable between the groups. Logarithmic transformation was tested for time-related variables regarding the best fit of time. A *p*-value <0.05 was considered statistically significant (SAS 9.4, Cary, NC, the United States).

## Results

### Characteristics of EEG seizures and spikes/sharp waves

Postoperative EEG seizures occurred in 8.3% (28 out of 337) patients. There were 309 patients without seizures (Group N), 15 with secondarily generalized seizures (Group G), and 13 with focal seizures (Group F).

**Figure 2:**
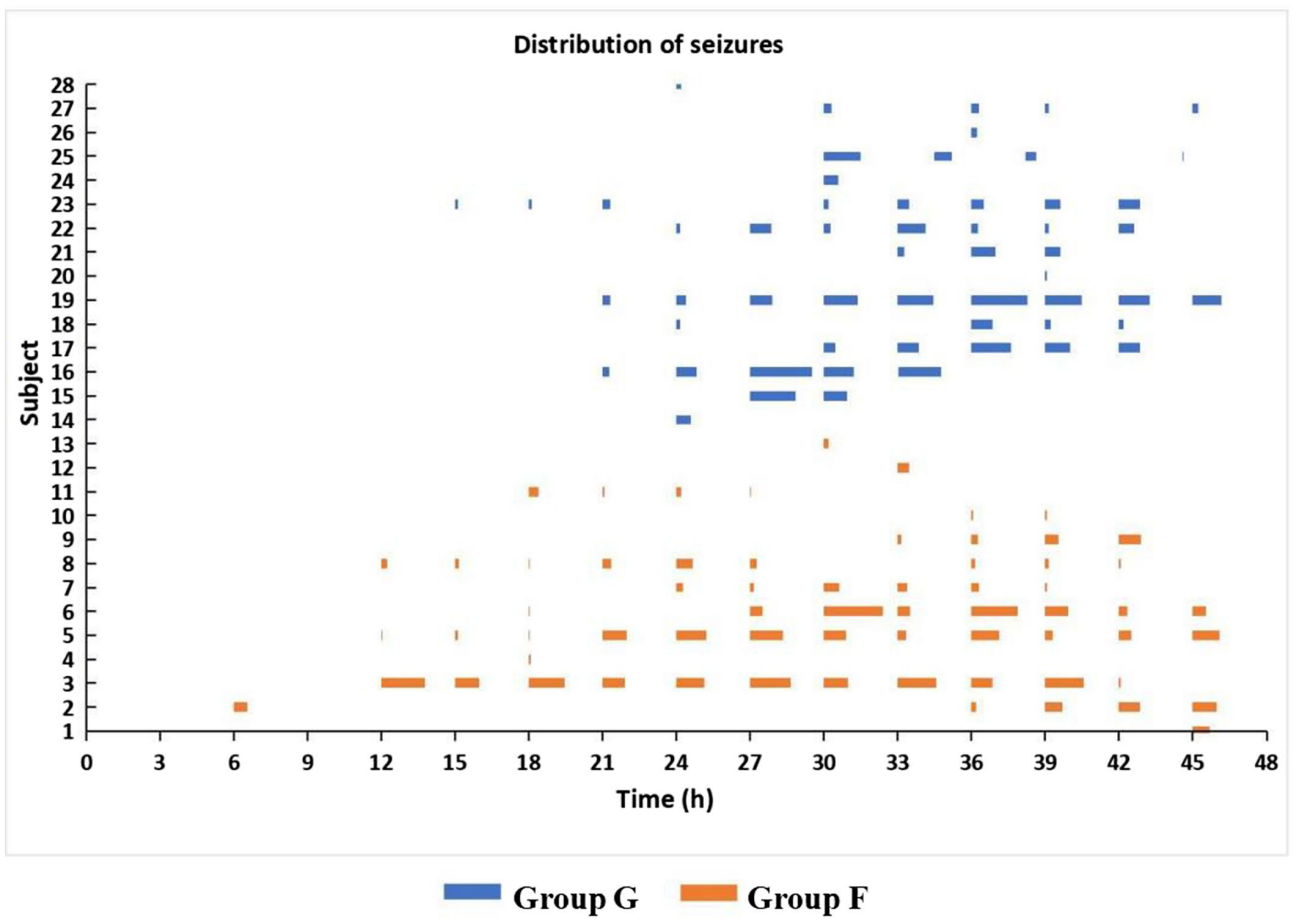
Time distribution of seizures in secondarily generalized seizure group (Group G) and focal seizure group (Group F) during the 48 h study period after CPB. *Ps* ≥ 0.27 for the onset time and duration of seizures and the incidence of status epilepticus.

**Figure 3:**
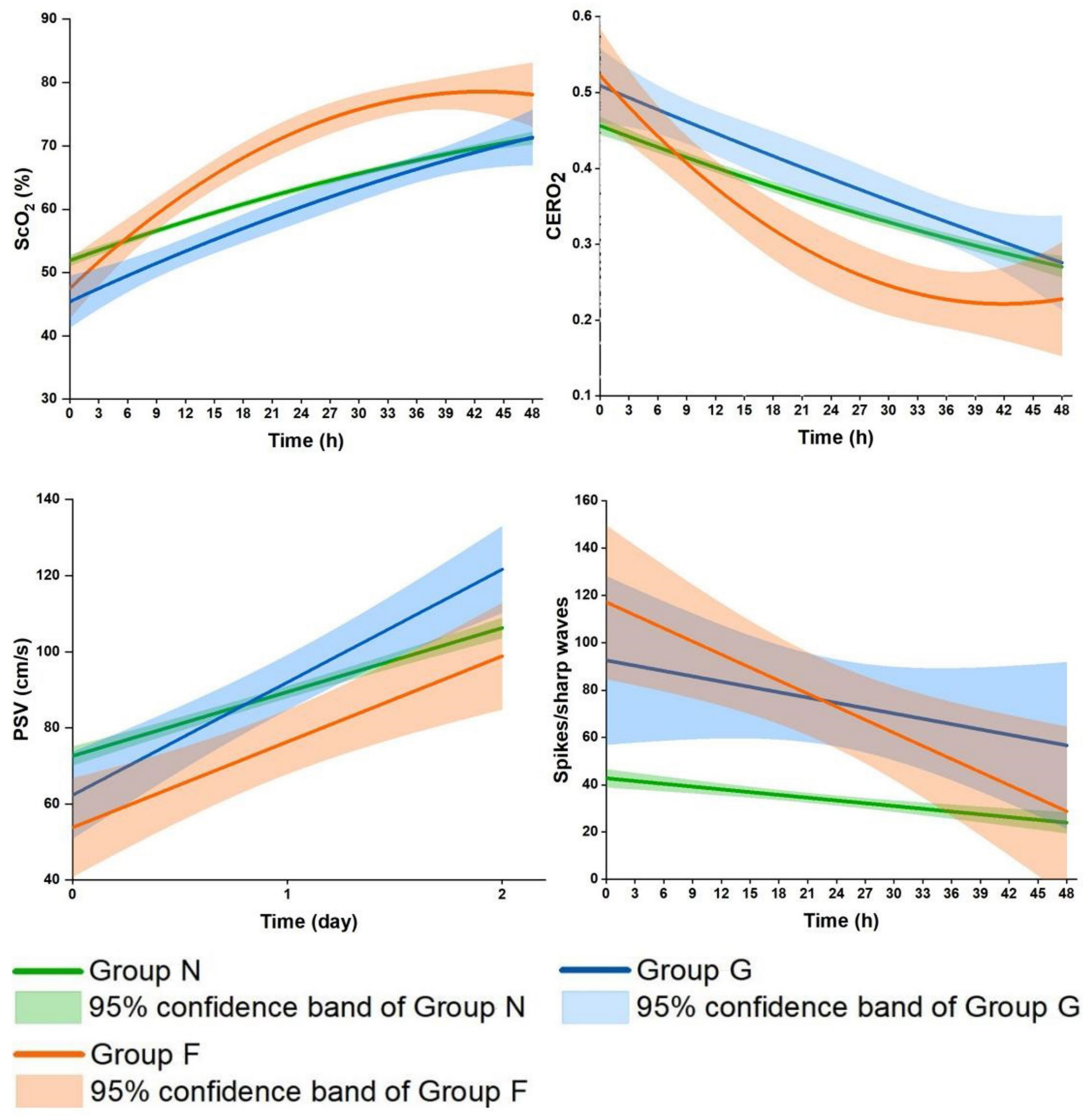
Comparisons of the profiles of the cerebral oxygenation parameters and spikes/sharp waves during the first 48 h after CPB among the three groups. Group G, secondarily generalized seizures group; Group F, focal seizures group; Group N, none-seizures group; ScO_2_, cerebral oxygen saturation; CERO_2_, cerebral oxygen extraction ratio; PSV, peak systolic velocity.

In Group G, the onset time of seizures ranged from 15–39 h (median 27) after surgery and lasted 1.6–646.8 min (median 84.9). Among them, 11 (73.3%) had status epilepticus. Seizure onset originated from occipital regions in 6 (40.0%), frontal in 2 (13.3%), and lateralized in 7 (46.7%). In Group F, the onset time of seizures ranged from 6 to 45 h (median 18) after surgery and lasted 3.6–772.9 min (median 106.3) and 10 (76.9%) had status epilepticus. Seizure onset originated from occipital regions in 8 (61.5%), central in 4 (30.8%), and frontal in 1 (7.7%). Seizures were lateralized in 1 (7.7%) and focal in 12 (92.3%). There were no significant differences in the onset time and duration of seizures and the incidence of status epilepticus (*Ps* ≥ 0.27) between the two seizures groups (Figure 2). The number of spikes/sharp waves was significantly larger (*P_group_* < 0.0001) and decreased significantly faster (P_group*time_ = 0.02) across Group N, Group G, and Group F (Table 2, Figure 3 and Supplementary Table S1).

**Table 2:** Statistical results of the comparison of the changes of cerebral oxygenation parameters and spikes/sharp waves during the first 48 h after cardiac surgery among the three groups.

Variables	Entire cohort					
	Time		Group		Group*Time	
	Parameter estimate	*p* value	Parameter estimate	*p* value	Parameter estimate	*p* value
ScO_2_ (%)	0.44	<0.0001	−1.35	0.30	0.12	<0.0001
CERO_2_	−0.004	<0.0001	0.02	0.23	−0.001	0.001
PSV (cm/s)	0.86	<0.0001	−9.19	0.003	0.27	0.0003
Spikes/sharp waves	−0.50	<0.0001	36.08	<0.0001	−0.30	0.02

ScO_2_, cerebral oxygen saturation; CERO_2_, cerebral oxygen extraction ratio; PSV, maximum peak systolic velocity.

### Demographic and clinical data among the three groups

Across Group N, Group G, and Group F, patients’ age, weight, and body surface area (BSA) trended to be lower (*Ps* ≤ 0.10); STS-EACTS Mortality Categories was significantly higher and the durations of CPB and ACC were significantly longer and the use of DHCA was more frequent (*Ps* ≤ 0.001). There were no significant differences in the durations of DHCA, postoperative mechanical ventilation, CICU, and hospital stay (*Ps* ≥ 0.07) among the three groups. There were 8 deaths in Group N, and none in Group G and Group F (*P* > 0.99) (Table 1).

### Postoperative profiles of cerebral oxygenation parameters in the three groups

ScO_2_ significantly increased in the three groups during the 48 h (Supplementary Table S1). The increase was significantly faster across Group N, Group G and Group F (*P_time_* < 0.0001, *P_group_* = 0.30, P_group*time_ < 0.0001). In more details, it linearly increased in Group N and Group G during this period (*Ps* < 0.0001); In Group G, it reached a level close to that in Group N by the end of 48 h; In Group F, it was significantly related to time after logarithmic transformation, with a faster increase followed by a slower increase (*P* < 0.0001), surpassing the level in Group N since 6–9 h after CPB. Similar but reciprocal profiles occurred in CERO_2_ (*P_time_* < 0.0001, *P_group_* = 0.23, P_group*time_ = 0.001). The overall levels of PSV were significantly lower during the 48 h (*P_group_* = 0.003), and increased more rapidly across Group N, Group G and Group F (P_group*time_ = 0.0003) (Table 2 and Figure 3). Further in Group F, there were no significant differences in ScO_2_, CERO_2_, and PSV between the two sides of the brain (*Ps* ≥ 0.17).

### Postoperative brain injuries on MRI in the three groups

Postoperative MRI was undertaken in 65.3% (220 out of 337) of patients including 199 (64.4%) in Group N, 11 (73.3%) in Group G, and 10 (76.9%) in Group F (*P* = 0.52) (Table 3). There were no significant differences in the types (hemorrhage, white matter injury, and stroke) and degrees of brain injury between Group G and N. Compared to Group N and Group G, Group F had the most severe degree of brain injury (*P* = 0.002). All the patients in Group F had brain injury, being mild, moderate, and severe in 6, 1, and 3, respectively. Among the types of brain injury, the incidence of white matter injuries (50.0%) was highest in Group F (*P* = 0.0002). Another 4 (40%) patients had hemorrhage (subarachnoid in 1, subdural in 3), and 1 (10%) patient had stroke (Table 3).

**Table 3:** Brain injuries on MRI in the three groups of patients.

Variable	Group N	Group G	Group F	*p*-value
The number of MRI	199 (64.4%)	11 (73.3%)	10 (76.9%)	0.52
Type of brain injury				
Hemorrhage	105 (52.8%)	7 (63.6%)	8 (61.5%)	0.19
White matter injuries	9 (4.5%)	0 (0.0%)	5 (50.0%)	0.0002
Stroke	5 (2.5%)	0 (0.0%)	1 (10.0%)	0.27
Degree of brain injury				0.002
Normal	87 (43.7%)	4 (36.4%)	0 (0.0%)	
Mild	101 (50.8%)	6 (54.6%)	6 (60.0%)	
Moderate	5 (2.5%)	1 (9.1%)	1 (10.0%)	
Severe	6 (3.0%)	0 (0.0%)	3 (30.0%)	

MRI, magnetic resonance imaging.

## Discussion

The present study demonstrated distinct profiles of cerebral oxygenation parameters in Group G and particularly Group F compared to Group N, potentially attributable to the dynamic and complex relations between cerebral metabolism, oxygen consumption, and its delivery in the different types of seizures.

The early hours after CPB represent the most critical period of imbalanced cerebral oxygenation with decreased cerebral oxygen delivery and increased oxygen consumption, as indicated by the initially low ScO_2_ and PSV and high CERO_2_ in our cohort. In the two groups with seizures compared to Group N, the initial ScO_2_ and PSV were lower, and CERO_2_ higher, indicating a poorer balance of cerebral oxygenation (12, 16, 17, 25, 26). Clinically, patients with seizures were sicker, with significantly higher STS-EACTS Mortality Categories, longer time of CPB, ACC, and more frequent use of DHCA, which have been identified as risk factors for seizures in our previous study and others (1, 2, 4, 5).

Nonetheless, it should be noted that seizures did not occur during the most critical period until the median time of postoperative 27 and 18 h (*P* = 0.27) in Group G and Group F, respectively. This finding is consistent with other studies (2, 4, 6). Instead of seizures, the abnormal discharges were mostly manifested as micro-scale patterns, i.e., spikes/sharp waves in the two seizure groups, particularly in Group F. Speculatively, cerebral oxygenation may have too limited capacity to manifest seizures (9). A study using phosphorus-31 magnetic resonance spectroscopy in infants with seizures has revealed that high-energy phosphates decrease by 33% and mitochondrial oxidative phosphorylation increases by 45% during seizures, indicating a depleted cerebral energy state (8).

Later, seizures occurred and spikes/sharp waves decreased as cerebral oxygenation status recovered to a certain degree, as indicated by the increase in ScO_2_ and decrease in CERO_2_. In the two seizure groups, these changes were greater compared to Group N. This seems puzzling, as seizures induce an increase in cerebral oxygen consumption which would expectedly lead to poor cerebral oxygenation status (10, 15). Furthermore in Group G, ScO_2_ gradually and linearly increased, CERO_2_ decreased, and both became close to the levels in Group N by 48 h. In Group F, the increase in ScO_2_ and decrease in CERO_2_ were related to time after logarithmic transformation, with a faster increase/decrease followed by a slower increase/decrease. The changes of the two parameters were so much faster that they surpassed the levels in Group N since postoperative about 6–9 h coinciding with the onset of seizures.

The distinct profiles in ScO_2_ and CERO_2_ may indicate different underlying pathophysiological mechanisms between the two types of seizures. In Group G, the secondarily generalized seizures are resulted from hyperexcitability and hypersynchrony of multiple neurons all over the brain (27, 28). The potentially increased cerebral oxygen consumption may be overcompensated by the greater increase in oxygen delivery. This was supported by our data showing a greater increase in PSV on both hemispheres on the 1st and 2nd postoperative day. Nonetheless, ScO_2_ remained lower, and CERO_2_ higher, until the end of 48 h study period, indicating a generally worse cerebral oxygenation status in Group G compared to Group N. Sokol et al. reported the lower level of ScO_2_ during the ictal phase of secondarily generalized seizures compared to pre-ictal baseline in adults with medically refractory epilepsy (28). Despite all this, there was no significant difference in brain injuries on MRI between Group G and Group N. The most frequent brain injury was subdural hemorrhage (mild brain injuries) in both groups (*n* = 7 (63.6%) in Group G and *n* = 105 (52.8%) in Group N), and some patients did not show brain injuries on MRI (*n* = 4 (36.4%) and *n* = 87 (43.7%), respectively). Seizures are related to functional neurologic impairment and cause excitotoxic injury, which may not respond or lead to structural abnormalities observed on MRI (10).

Group F may represent a more complex scenario in terms of cerebral oxygenation alteration. The greatest increase in ScO_2_ and decrease in CERO_2_ occurred in the presence of persistently lower PSV. These data might suggest a greater reduction in cerebral oxygen consumption relative to the reduced oxygen delivery. Furthermore, there were the greatest amount of spikes/sharp waves during the 48 h in Group F, likely indicating overall worse EEG discharge abnormalities. The findings on MRI may provide more insights into the potential underlying mechanisms of this distinct cerebral oxygenation alteration. Group F had the most severe brain injury on MRI compared to Group G and Group N. In fact, all the patients with focal seizures had positive MRI findings, half of them had white matter injuries and the other half had hemorrhage or stroke. In consistency with our data, patients with focal seizures caused by febrile were more likely to have abnormal white matter signals and subcortical focal hyperintensity (29–31). The widespread seizure activity largely relies on the neuron networks of distinct neural circuits of transverse fibers in the white matter and spreads to more distant areas via the corpus callosum (32). Thus white matter injuries may disrupt that transmission pathway, which may help to explain our findings in patients with white matter injury in Group F. The other 40% of patients in Group F had hemorrhage (subarachnoid in 1, subdural in 3). In fact, the incidence of hemorrhage was not significantly different among the three groups. Patients with brain hemorrhage developed focal or secondarily generalized seizures, which may be determined by the different excitabilities across the neuron network in different patients (33). Furthermore, the network inhibition hypothesis suggests that seizure activity in one part of the brain may cause inhibition of other cortical regions (34). As such, the reasons for the findings in Group F might appear clear. The majority of focal seizures onset originated from the occipital region (61.5%) and central region (30.8%) thereby inhibiting the frontoparietal regions (35, 36). While focal seizures may increase oxygen consumption focally (28, 35, 37) we placed the NIRS sensors in the frontal regions and measured PSV in the middle cerebral artery where cerebral metabolism and oxygen consumption were inhibited. The inhibition of cerebral oxygen consumption was so much that the greatest increase in ScO_2_ and decrease in CERO_2_ occurred even when PSV remained consistently lowest in Group F compared to Group G and Group N. The inhibition appeared to have involved both hemispheres as there were no significant differences in ScO_2_, CERO_2_, and PSV on the two sides of the brain.

### Strengths and limitations

The main strength of this study was that we conducted a comprehensive and systematic investigation using NIRS, TCD, EEG, and MRI to evaluate cerebral oxygenation alteration in relation to brain injury. While ScO_2_ is widely used during and after CPB, there is limited knowledge about how to interpret the ScO_2_ values. Previous studies have mostly focused on reduced ScO_2_ (18–20). Our data provided information about the dynamic changes in ScO_2_ along with other cerebral oxygenation parameters in the complex relation to varied abnormal EEG discharges and brain injury on MRI. This information may be helpful to interpret the routinely monitored data at bedside. A state of ‘overshooting’ ScO_2_ with persistently low PSV was more frequently seen in those with focal seizures and more severe brain injury. More attention should be paid to the clinical management in such patients to protect their brain. This study has limitations. (1) This was a single-center study that recruited heterogeneous and relatively more complex CHD patients. Our data may not be applied to the overall patient populations in our center or other patient populations such as neonates with hypoplastic left heart syndrome and related anomalies who are known to have the most severe brain injuries (1, 38). (2) The STS-EACTS Mortality Categories was updated in 2021 (39) which was near the end of our study. We decided to keep using the earlier version throughout the study period in order to avoid certain bias that exists between the two versions. The information obtained in our study remained valid. (3) The mechanisms for the altered cerebral oxygenation in different groups were speculated based on previous animal and human experimental findings. (4) The effect of varied EEG discharge abnormalities and cerebral oxygenation status on long-term neurodevelopmental outcomes remains to be explored, which is being investigated in our center.

## Conclusion

Distinct profiles of cerebral oxygenation parameters were found in patients with secondarily generalized or focal seizures in children after CPB compared to patients without seizures, which was potentially attributable to the dynamic and complex relations between cerebral oxygen consumption and its delivery in the different types of seizures. A state of ‘overshooting’ ScO_2_ with persistently low PSV was more frequently seen in those with focal seizures and more severe brain injury. More attention should be paid to the clinical management in such patients to protect their brain. Information from this study may have important clinical implications in detecting brain injuries when monitoring cerebral oxygenation in this vulnerable group of children after CPB.

## Data availability statement

The raw data supporting the conclusions of this article will be made available by the authors, without undue reservation.

## Ethics statement

The studies involving humans were approved by Guangzhou Women and Children’s Medical Center. The studies were conducted in accordance with the local legislation and institutional requirements. Written informed consent for participation in this study was provided by the participants’ legal guardians/next of kin.

## Author contributions

RL: Data curation, Investigation, Methodology, Validation, Writing – original draft, Writing – review & editing. ND: Data curation, Methodology, Resources, Validation, Writing – review & editing. SN: Data curation, Resources, Validation, Writing – review & editing. MZ: Data curation, Resources, Validation, Writing – review & editing. JF: Data curation, Validation, Writing – review & editing. XC: Resources, Supervision, Validation, Visualization, Writing – review & editing. LM: Investigation, Resources, Supervision, Validation, Writing – review & editing. JL: Methodology, Project administration, Resources, Supervision, Visualization, Writing – original draft, Writing – review & editing.
