# Supplementary material for: Distinct profiles of cerebral oxygenation in focal vs. secondarily generalized EEG seizures in children undergoing cardiac surgery
Source: Front Neurol. 2024 May 9;15:1353366. doi: 10.3389/fneur.2024.1353366 (PMC11111896; doi:10.3389/fneur.2024.1353366)
Supplement: Supplementary file 1 [file Table_1.DOCX]

Supplemental 1. Statistical analysis of the changes of cerebral oxygen in relation to time during 48 hours after cardiac surgery in the three groups.

|  | Group N | | |  | Group G | | |  | Group F | | |
| --- | --- | --- | --- | --- | --- | --- | --- | --- | --- | --- | --- |
| Variables | Intercept | Parameter estimate | P values |  | Intercept | Parameter estimate | P values |  | Intercept | Parameter estimate | P values |
| ScO_2_ (%)* | 52.68 | 0.44 | <.0001 |  | 46.71 | 0.53 | <.0001 |  | 28.23 | 13.86 | <.0001 |
| CERO_2_* | 0.45 | -0.004 | <.0001 |  | 0.51 | -0.005 | <.0001 |  | 0.46 | -0.006 | <.0001 |
| PSV (cm/second) | 73.99 | 0.85 | <.0001 |  | 65.32 | 1.40 | <.0001 |  | 55.57 | 1.15 | <.0001 |
| Spikes/sharp waves | 44.89 | -0.52 | <.0001 |  | 75.79 | -0.008 | 0.99 |  | 119.56 | -1.59 | 0.003 |

Note. *Data were entered after logarithmic transformation in Group F.

*ScO2* = cerebral oxygen saturation, *CERO_2_* = cerebral oxygen extraction ratio, *PSV* = maximum peak systolic velocity.
